# Recombination elevates the effective evolutionary rate and facilitates the establishment of HIV-1 infection in infants after mother-to-child transmission

**DOI:** 10.1186/s12977-015-0222-0

**Published:** 2015-11-16

**Authors:** Keri B. Sanborn, Mohan Somasundaran, Katherine Luzuriaga, Thomas Leitner

**Affiliations:** Program in Molecular Medicine, University of Massachusetts Medical School, 373 Plantation Street, Worcester, 01605 MA USA; Theoretical Biology and Biophysics, Los Alamos National Laboratory, Los Alamos, 87545 NM USA

**Keywords:** HIV-1, MTCT, Transmitted/founder virus, Recombination, Adaptation, Evolutionary rate

## Abstract

**Background:**

Previous studies have demonstrated that single HIV-1 genotypes are commonly transmitted from mother to child, but such analyses primarily used single samples from mother and child. It is possible that in a single sample, obtained early after infection, only the most replication competent virus is detected even when other forms may have been transmitted. Such forms may have advantages later in infection, and may thus be detected in follow-up samples. Because HIV-1 frequently recombines, phylogenetic analyses that ignore recombination may miss transmission of multiple forms if they recombine after transmission. Moreover, recombination may facilitate adaptation, thus providing an advantage in establishing infection. The effect of recombination on viral evolution in HIV-1 infected children has not been well defined.

**Results:**

We analyzed full-length *env* sequences after single genome amplification from the plasma of four subtype B HIV-1 infected women (11–67 *env* clones from 1 time point within a month prior to delivery) and their non-breastfed, *intrapartum*-infected children (3–6 longitudinal time points per child starting at the time of HIV-1 diagnosis). To address the potential beneficial or detrimental effects of recombination, we used a recently developed hierarchical recombination detection method based on the pairwise homoplasy index (PHI)-test. Recombination was observed in 9–67 % of the maternal sequences and in 25–60 % of the child sequences. In the child, recombination only occurred between variants that had evolved after transmission; taking recombination into account, we identified transmission of only 1 or 2 phylogenetic lineages from mother to child. Effective HIV-1 evolutionary rates of HIV-1 were initially high in the child and slowed over time (after 1000 days). Recombination was associated with elevated evolutionary rates.

**Conclusions:**

Our results confirm that 1–2 variants are typically transmitted from mothers to their newborns. They also demonstrate that early abundant recombination elevates the effective evolutionary rate, suggesting that recombination increases the rate of adaptation in HIV-1 evolution.

**Electronic supplementary material:**

The online version of this article (doi:10.1186/s12977-015-0222-0) contains supplementary material, which is available to authorized users.

## Background

Recombination contributes significantly to intrapatient HIV-1 diversity [1–3]. HIV-1 recombination is a complex phenomenon defined by both the template switching rate of the viral reverse transcriptase and the probability of coinfection of a single host cell [4, 5]. Estimates of the effective recombination rate based on longitudinal studies of patient samples range from 1.4 ± 0.6 × 10^−5^ to 1.38 × 10^−4^ recombinations per site per generation [3, 5], and over time recombinants may accumulate in as many as 1 out of 3 *env* sequences [6]. While widespread among many organisms, including eukaryotes, bacteria and viruses, the role of recombination is still debated [7–10]. Clearly, recombination can bring beneficial alleles together, increasing the fitness of the recombinant, but recombination may also separate beneficial alleles that occur on the same genome. Theoretical studies have shown that the benefit of recombination depends on factors such as the interplay of recombination and substitution rates, population size, and epistatic interactions [11–17]. However, the lack of direct evidence of whether recombination is beneficial or detrimental calls for additional studies, where rapidly evolving, recombining retroviruses may provide a good model system.

HIV-1 sequences observed within the first few months of mucosal transmission are typically homogeneous due to the transmission bottleneck [18–21], subsequently diverging and diversifying over the course of infection [22–24]. Studies have shown an inverse relationship between the rate of viral evolution and disease progression [25–30], including in vertically infected infants [26], likely due to immune selective pressures. Importantly, recent theoretical work suggested that recombination may accelerate HIV-1 adaptation [17]. While the role of recombination in the establishment of HIV-1 infection is unknown, evolutionary theory suggests [11–16] that recombination may alter the genetic variation upon which natural selection can operate. This would thus likely increase the overall evolutionary rate. Additionally, because simple phylogenetic analysis ignores recombination by assuming single parents of each lineage, frequent recombination of HIV-1 within an individual could also potentially mask the transmission of multiple variants. Thus, because recombination may be fundamentally important to HIV-1 infection and evolution, and because recombination may interfere with standard phylogenetic analyses, it is important to consider potential recombinants in the assessment of HIV-1 transmission.

Mother-to-child HIV-1 transmission (MTCT) is the primary mode of pediatric infection, accounting for up to 16 % of all HIV-1 transmission events globally [31]. While combination anti-retroviral (ARV) regimens can effectively reduce MTCT, the use of ARV is limited by cost and logistical requirements in limited-resource settings. Most studies evaluating MTCT specifically have been limited to one time point post-transmission. However, the possibility remains that additional variants are transmitted that do not execute the infection or post-transmission steps as efficiently as those detected early in infection. Although present at a low level early in infection, these variants could later expand and contribute to the course of infection. Such variants may also recombine with more frequent variants, and therefore not be detected with simple phylogenetic methods. Thus, evaluating longitudinal samples is critical to determining whether additional variants may be transmitted but missed during early sampling.

In this study, we evaluated HIV-1 *env* sequences obtained from samples collected longitudinally from four mother–child pairs (1) to evaluate what effects recombination may have on HIV-1 transmission and the subsequent evolutionary rate, and (2) to determine the number of variants transmitted from mother to child. We found high proportions of recombinant HIV-1 forms in both the mothers and their infants and that recombination elevates the effective evolutionary rate. Taking recombination into account, we identified transmission of 1 or 2 phylogenetic lineages from mother to child. Recombination only occurred between variants that evolved after transmission; recombinant forms thus did not hide multiple transmitted maternal HIV-1 variants. Our results show that recombination occurs frequently and appears to contribute significantly to the early diversification of the viral quasispecies in infants.

## Results

### Widespread recombination in mother and child HIV-1 populations

**Fig. 1:**
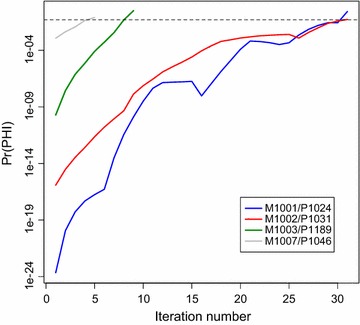
Progress of h-PHI removing recombinant taxa among mother–child sequences. The h-PHI test removed the sequence contributing most to the PHI signal in each iteration, recalculating the overall PHI score after each removal as the homoplasy relationships changed depending on the current sample. Datasets with many recombinants started at a lower Pr(PHI) value and had more taxa removed (Table 1). The *colored lines* show the iterative progress, and the *dashed line* indicates Pr(PHI) = 0.05, above which the iterative removal process stopped

Initial phylogenetic analyses of the *env* sequences from the four mother–child pairs showed low statistical support for many clades, and dividing the long sequences into five fragments (~500 nt each) showed that the phylogeny across those fragments was not stable (data not shown). To assess whether this instability could be explained by recombination, we applied a recently developed hierarchical test based on the PHI-test [32, 33], henceforth referred to as h-PHI. Because apolipoprotein B mRNA editing enzyme, catalytic polypeptide-like (APOBEC)-induced hypermutation [34, 35] may cause homoplasies that may mislead recombination detection, we first removed hypermutants from the data; three hypermutant sequences were detected and removed in child P1024 (P1024-1B, P1024-7A, P1024-2B). The h-PHI test showed that both the mother and the child HIV-1 populations carried many recombinants (Table 1). The h-PHI test iteratively removed recombinant taxa until the remaining set of mother and child sequences indicated at Pr(PHI) >0.05 that no recombination signal remained (Fig. 1). In the mother–child pairs M1001/P1024 and M1002/P1031, about half of all of the sequences detected were recombinants according to h-PHI (Table 1). M1003/P1189 and M1007/P1046 had fewer recombinants detected; this can be explained by an overall lower diversity, which should make detection less powerful. Note that when the ancestral sequences of the recombinant are genetically similar, there should be less of a biological impact of recombination, and thus the detection ability should to some degree follow the general genetic diversity in the recombining population (the fraction of h-PHI-detected recombinants compared to the population diversity in all eight patients showed a Pearson’s product-moment correlation of 0.622, p = 0.099).

**Table 1:** Recombination detection results of the h-PHI test

MTCT pair	N_seq_	N_hyper_	Pr(PHI_all_)	Pr(PHI_end_)	N_recomb_	Diversity	Recomb (%)
M1001/P1024	18/38	0/3	2.23e−24	0.269	12/23	15/25	67/60
M1002/P1031	25/56	0/0	1.21e−16	0.053	15/26	13/23	60/46
M1003/P1189	11/31	0/0	1.93e−10	0.321	1/12	5/26	9/39
M1007/P1046	22/67	0/0	1.17e−3	0.079	2/17	5/14	9/25

*N*_*seq*_ number of sequences investigated, *N*_*hyper*_ number of hypermutants detected, *Pr(PHI*_*all*_ *)* probability of PHI-test with all non-hypermutant sequences, *Pr(PHI*_*end*_ *)* probability of PHI-test with recombinant sequences removed at the end of the h-PHI iterations, *N*_*recomb*_ number of recombinant found with the h-PHI algorithm, *Diversity* mean pairwise distance among all non-hypermutated taxa measured in 10^−3^ substitutions/site using a F84 substitution model, *Recomb* (%) percent recombinants found out of all sequences

**Fig. 2:**
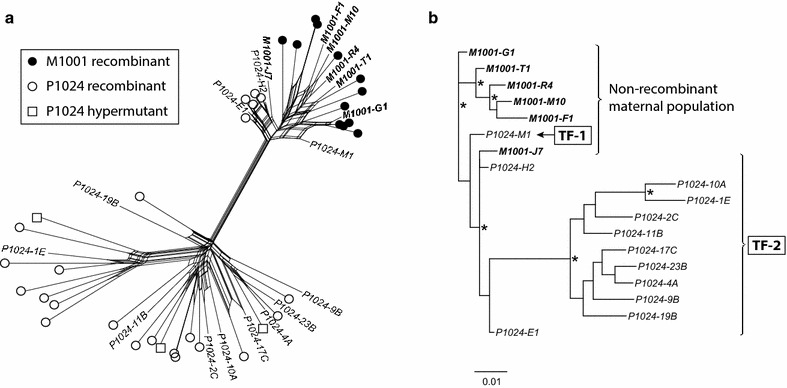
Removing recombinant taxa stabilized the reconstructed phylogeny of mother–child transmission. SplitsTree inference of mother–child pair M1001/P1024 (**a**) and the corresponding dichotomous phylogeny of the non-recombinant taxa after removal of the h-PHI-detected recombinant taxa (**b**). Maternal non-recombinant taxa are labeled with *bold* text names, and child non-recombinant taxa are labeled with *plain* text names. Recombinant taxa are labeled by *filled* (maternal) or *unfilled* (child) *circles*. *Unfilled squares* denote hypermutated taxa. *Stars* in the phylogeny (*panel* **b**) indicate robust cladistic support (aLRT > 0.90). The deduced transmitted forms (TF-1 and TF-2) are indicated in the phylogeny. The *scale bar* in the phylogeny is in units of substitutions/site

Removing hypermutant and recombinant sequences from the populations resulted in phylogenies with more stability. Figure 2 shows the result for pair M1001/P1024 (data from all pairs are shown in Additional file 1: Figure S1). A SplitsTree analysis clearly shows the many alternative relationships between the taxa caused by the multiple ancestries of the recombinant taxa. When the recombinants detected by our h-PHI test were removed (as were hypermutants), enough stability was achieved in the remaining phylogeny to assess how many separate phylogenetic lineages must have established the child population (Additional file 1: Figure S1). Based on non-recombinant taxa only, P1024 was infected by two separate lineages from M1001, and the other children (P1031, P1189, P1046) were all infected by a single maternal HIV-1 lineage.

### Recombination does not hide multiple transmitted taxa

**Fig. 3:**
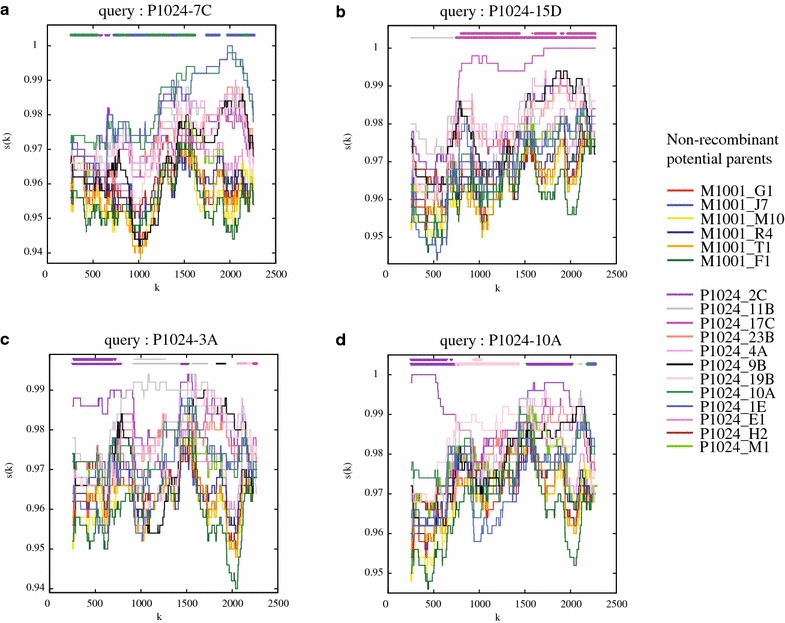
Typical examples of RIP analyses of h-PHI-identified recombinant taxa. These examples come from M1001/P1024 and are representative of all RIP results among all four mother–child pairs investigated in this study (Additional file 2: Table S1). *Panel* **a** shows a result where the query sequence (h-PHI-identified recombinant taxon P1024-7C) does not have a significant similarity with any one detected non-recombinant maternal or child sequence in any part of the sequenced genome (2500 nt of *env*). *Panel* **b** shows an example where the h-PHI-identified recombinant taxon P1024-15D has one parent (P1024-17C) significantly more similar in part of the sequence (approximately nucleotide sites k = 700–2500) and likely one missing parent (sites 1–700) in the detected set of non-recombinant potential parents. *Panels* **c** and **d** show examples of results where >1 parent shows significant similarity to some part of the query sequence (P1024-3A to P1024-2C and P1024-11B, and P1024-10A to P1024-2C and P1024-19B). These latter two *panels* also show the situation where no parent is significantly more similar than the other potential parents because several are fairly similar (sites 1500–2500). The similarity, s(k), to each non-recombinant potential parent is displayed as a *colored line*, identified in the list to the *right* of the RIP result panels. Each RIP result also shows the best match in the corresponding *color* as the *lower horizontal bar* in the *top* of each *panel*, and if the match is significant, another *colored bar* on *top* of that is shown for the corresponding sites

While the results from the non-recombinant taxa suggested 1 or 2 transmitted maternal HIV-1 lineages, it remained possible that several maternal lineages were transmitted, which subsequently recombined in the child and died out before sampling (or only existed at levels below the detection limit of our sequencing system), leaving only the trace of multiple transmitted forms in the recombinant taxa. To evaluate this possibility, we tested whether any of the h-PHI-identified recombinants were chimeric between >1 maternal ancestors, or if recombination only involved ancestral taxa from within the child’s own HIV-1 population using “Recombination Identification Program” (RIP) analysis, which calculates the similarity of a recombinant sequence to potential ancestral sequences in a sliding window analysis. Figure 3 shows examples of the RIP analyses of P1024 h-PHI identified recombinant taxa. Three different types of patterns were typically observed: (1) no significant similarity to any potential available ancestral sequences; (2) partial significant similarity to 1 available ancestor; and (3) partial significant similarity to >1 available ancestor. The instances in which RIP could not find significant similarity, partially or fully, are explained by two factors: (1) lack of non-recombinant ancestor, i.e., the ancestor was not detected in our set of sequences (not available to our analysis), and (2) all ancestors were so similar that no single sequence was significantly more similar to the recombinant than the others. These three patterns occurred in each of the children’s HIV populations (Additional file 2: Table S1). Importantly, neither in P1024, nor in P1031, P1189 or P1046, did we detect a significant trace of multiple maternal ancestor sequences using RIP, and thus these recombinants did not hide additional transmitted forms beyond those detected among the non-recombinant taxa. In fact, all recombination among the child HIV-1 populations occurred in the child between lineages that had formed after transmission.

In summary, P1024 was infected by two maternal HIV-1 variants, while P1031, P1189 and P1046 were infected by 1 variant each from their respective mothers. While there was widespread recombination in all mothers and children, all recombination in the child HIV-1 populations occurred among lineages that had split off the transmitting form after transmission.

### Recombination boosts the HIV-1 effective evolutionary rate

As the HIV-1 population becomes established, the corresponding phylogeny grows in height as the phylogenetic lineages accumulate mutations. In reference to the mother’s HIV-1 population, the evolutionary rate in the child’s population generally slows down towards the tips of the tree (Table 2). All child HIV-1 populations evolved more slowly at the full tree height than closer to the root when the population was initiated (p = 0.008, paired Wilcoxon signed rank test). P1024 had a much higher apparent evolutionary rate than the other child populations, which may be explained by the fact that P1024 was infected with 2 HIV-1 variants from the mother’s population, which amplifies the apparent evolutionary rate due to the transmitted ancestral divergence [36, 37]. In P1189 and P1046, the evolutionary rates first increased as the trees grew (indicated by the range in Table 2) and then decreased to levels below any initial rate, hence following the general pattern in these HIV-1 populations. Importantly, recombination boosted the evolutionary rates across the tree height (Table 2).

**Table 2:** Evolutionary rates across tree height

Patient	With recombinants		Without recombinants	
	Initial rate	Final rate	Initial rate	Final rate
P1024	55.4	8.40	19.8	3.08
P1031	3.90	1.60	3.46	2.73
P1189	1.90–3.74	1.38	5.19	1.28
P1046	2.81–6.80	0.56	2.62–4.72	0.63

*Initial rate* rate at start of child HIV-1 subtree, *Final rate* rate at tips of child HIV-1 subtree. All rates are in units of 10^−5^ substitutions site^−1^ day^−1^ and were found at R^2^ > 0.90 and p < 0.001 by ordinary least squares regression

To investigate the evolutionary rate as a function of time, we divided the sampled time period on the middle sample (potential inflection point) and compared the resulting rates on each side (rates 1 and 2 in Table 3). Similar to the tree height, the evolutionary rate as a function of time also decreased after a high initial level (p = 0.016, paired Wilcoxon signed rank test). P1024 again showed much higher rates, again explained by transmission of >1 maternal variant. Notably, while recombination again inflated the evolutionary rates (p = 0.016, paired Wilcoxon signed rank test), the effect appeared to be less on the time scale than on the tree height level. With viral load data from only two of the transmission pairs, we could not observe any correlation between the dynamics of the evolutionary rate and viral load or antiviral treatment. There was also no clear pattern in our data that suggested that recombination was more common in earlier or later samples from the child.

**Table 3:** Evolutionary rates across sampling times

Patient	With recombinants		Inflection time (days)	Without recombinants	
	Rate 1	Rate 2		Rate 1	Rate 2
P1024	15.4	9.44	1163	4.38	1.88
P1031	3.95	1.84	1377	3.16	1.91
P1189	3.02	3.30	886	2.17	1.79
P1046	4.33	0.67	706	3.58	0.56

*Rate 1* rate before potential inflection point, *Rate 2* rate after potential inflection point, *Infection time* time of potential change in rate. All rates are in units of 10^−5^ substitutions site^−1^ day^−1^ and were found at R^2^ > 0.90 and p < 0.001 by ordinary least squares regression, except Rate 2 of P1024 with recombinants at R^2^ = 0.82

Overall, these results suggest that the initial evolution was rapid in order to quickly adapt to the new (child) host. As new mutations were established, the evolution gradually slowed down. This qualitative pattern was independent of whether recombinants were included or not, but importantly, recombination quantitatively elevated the evolutionary rates.

### Env sequence characteristics in mother–child pairs

Previous studies have demonstrated universally convergent features in transmitted variants compared to the sequences predominating in the transmitter, suggesting that these features may somehow offer an advantage during HIV-1 transmission. In addition to CCR5 (R5)-tropism, fewer N-linked glycosylation sites, and lower V3 region charge, additional motifs have been reported in the literature that have various functional effects, including increased Env density on virions, resistance to broadly neutralizing antibodies, increased coreceptor binding, and escape of cytotoxic T lymphocyte (CTL) recognition [19, 38–42]. To evaluate whether recombination brought together or separated such known sequence motifs, indicating a fitness advantage or disadvantage, respectively, we evaluated the computationally predicted co-receptor tropism, V-region length and charge, and number of potential N-linked glycosylation (PNG) sites, as well as the presence of potentially advantageous motifs, in the sequences obtained from the four mother–child pairs. In silico analysis of the Env sequences from our cohort indicated that all transmitted Envs were indeed R5-tropic, although one CXCR4 (X4)-tropic Env sequence was predicted in one of the children at a later time point (P1046, at 168.62 months, Additional file 3: Table S2). In order to simplify the analysis of sequence motifs, and to consider potentially disadvantageous motifs, e.g., PNGs that allow for stronger antibody binding, we analyzed the lack of such PNGs, as Envs lacking these motifs may have an advantage during selection. Counting the potentially advantageous form of each motif enabled us to sum together the number of such motifs in order to compare recombinant and non-recombinant sequences. The number of potentially advantageous motifs present in the Env sequences varied between the mother/child pairs. Recombinant sequences contained approximately the same numbers of these motifs as non-recombinant sequences, indicating that recombination did not result in the net accumulation or loss of the potentially advantageous motifs considered (Additional file 3: Table S2). The distribution of most motifs was similar between the maternal Env sequences and those in the child at the first time point, suggesting that these motifs were not preferentially transmitted from mother to child (Additional file 4: Table S3). It is possible that there are other, yet unidentified or outside *env*, motifs/alleles that may constitute fitness advantages during early HIV infection.

## Discussion

Analysis of full-length *env* sequences from HIV-1 infected women and their infants over multiple time points revealed extensive recombination. Moreover, the observed recombination was associated with an elevated effective evolutionary rate, suggesting that recombination of HIV-1 facilitates adaptation to the new host after transmission. It has been previously shown that recombination may lead to the rapid emergence of antiviral drug resistance [43–45]. Together, these results support the notion that recombination is advantageous for HIV-1 adaptation.

Taking recombination into account, the examination of longitudinal samples confirmed the transmission of just one variant in all but one infant. These results are consistent with the results of our previous work with this cohort [38], which demonstrated the transmission of one or two variants from mother to child in each pair.

Recombination is known to occur during HIV-1 replication [46, 47] and inter-subtype recombinants are both frequent and successful in the worldwide HIV-1 epidemic [48–50]. Since genetic variability is typically much smaller at the level of the individual host, intra-host (and intra-subtype) recombinants have been described but are more difficult to detect [4–6, 51]. While recombination can create additional genetic diversity upon which natural selection can rapidly select advantageous variants and purge deleterious mutations from a population [11–15], recombination may have costs and may impede adaptation due to multidimensional epistasis, e.g., when new alleles are beneficial only if they accumulate in a prescribed order [16]. Thus, theoretically, recombination can either bring beneficial alleles together or separate beneficial alleles that already occur on the same genome. The debate on why sexual reproduction exists has led some authors to question why RNA viruses recombine [10]. Addressing these theoretical issues, it was recently shown that recombination substantially increases the rate of adaptation among a wide range of population sizes and mutation rates [17]. Our results indicating that recombination elevated the evolutionary rate are supportive of the beneficial effect of recombination during the establishment of HIV-1 infection in a new host.

If recombinants have a fitness advantage, one would expect them to carry more advantageous alleles/motifs as a result of recombination joining them onto the same genome. In our analyses, recombinants did not contain higher proportions of computationally inferred sequence features such as co-receptor tropism, V-region length and charge, number of PNG sites, or other motifs that have been previously described in the literature as potentially advantageous in transmission or early infection. It seems likely that these are not the features that additively make genomes more fit during the early stages of infection. Rather, escaping additional cytotoxic T lymphocyte and antibody immune epitopes not captured by these features may drive the early selection of recombinants.

After the early increase in effective evolutionary rates, the children’s viruses displayed a decreasing HIV-1 evolutionary rate as both the tree height and time grew. This has also previously been described in HIV-1 infected adults [23]. The decreasing evolutionary rate may be explained by the fact that functional constrains may make it harder to find advantageous mutations once the initial mutations have been established in the new host [23]; decreasing pressure from the immune system associated with disease progression [23] and the introgression of HIV-1 from latent reservoirs [32] may also contribute.

The detection of high levels of recombination in maternal HIV-1 sequences suggests that recombination is not limited to the establishment of infection. As HIV-1 is relentlessly confronted by the adaptive immune system, HIV-1 accumulates mutations to evade recognition, leading to an inverse relationship between the rate of viral evolution and disease progression [25–30]. Thus, it is likely advantageous for HIV-1 to recombine throughout the entire infection period.

## Conclusions

Longitudinal data provide the advantage that many biological processes can be directly observed, rather than predicted by modeling from cross-sectional data. Here, we show that recombination is common and increased the effective evolutionary rate but did not obscure the transmission of multiple HIV-1 variants from mothers to their children. As in adults, the overall evolutionary rate decreased as HIV-1 infection matured in the children. Altogether, these data suggest a beneficial effect of recombination during the establishment of HIV-1 infection in a new host.

## Methods

### Patient materials

We studied four HIV-1 subtype B infected mothers and their infected infants (Table 1), who contracted HIV-1 at birth, based on standard diagnostic criteria [52]. *Env* sequences were isolated from the mothers and children using single-genome amplification (SGA) from plasma samples. Maternal samples for these analyses were obtained at or within a month of delivery, and infant samples were obtained from the time point of diagnosis as well as from several time points thereafter. The children were not breastfed, and thus the sequences obtained at later time points represent variation originating from the variant(s) existing in the mother before or at birth.

### Ethics statement

All studies involving human samples were approved by the University of Massachusetts Medical School Institutional Review Board for Human Subjects. Informed, written consent was obtained from each of the women for their own and their infants’ participation prior to enrollment in this study.

### PCR amplification and DNA sequencing of HIV-1 *env*

Full-length HIV-1 *env* was amplified from RNA isolated directly from plasma by endpoint dilution nested reverse transcription polymerase chain reaction (RT-PCR), as previously described [38]. The outer and inner primer pairs, flanking the N- and C-termini of the *env* sequence (HXB2 positions 5948-8916) were the same as reported by Wei et al. [53]. The ~3 kb *env* amplicons were gel purified using the QIAquick Gel Extraction kit (QIAgen, Valencia, CA, USA) and sub-cloned into the pcDNA3.1/V5-His TOPO TA vector (Invitrogen Life Technologies, Carlsbad, CA, USA), then used to transform OneShot Stbl3 chemically competent *E. coli* (Invitrogen) following the manufacturer’s instructions. Colonies containing full-length inserts in the correct orientation were identified and screened for functionality using a syncytia assay, as previously described [38]. Each functional clone obtained from each subject arose from an independent endpoint dilution PCR.

The full-length gp160 of all viable molecular *env* clones were sequenced using BigDye Terminator chemistry. Sequences were assembled and trimmed using Geneious version R6 (Biomatters Ltd., Auckland, New Zealand). The sequences are available from GenBank under nucleotide sequence accession numbers KT283686–KT283953.

### *Env* sequence characteristics analyses

*Env* sequences from each subject were aligned using Geneious. The V region length, charge, and number of potential N-linked glycosylation sites (PNGs) were determined using the Variable Region Characteristics calculator on the Los Alamos National Laboratory website (http://www.hiv.lanl.gov/content/sequence/VAR_REG_CHAR/) using HXB2 as a reference sequence. Coreceptor tropism (CCR5 vs. CXCR4) was predicted in silico using the WebPSSM tool (http://indra.mullins.microbiol.washington.edu/webpssm/).

### Phylogenetic analyses

For our phylogenetic analyses, all MTCT pairs were aligned separately using MAFFT under the FFT-NS-i algorithm [54], and the alignments were codon corrected using GeneCutter [55] and manually checked using SeaView [56].

Hypermutants, i.e., dead-end HIV-1 variants with excessive A-to-G mutations induced by the host APOBEC mechanism [34, 35], were identified and removed using Hypermut (http://www.hiv.lanl.gov/content/sequence/HYPERMUT/hypermut.html) with the default APOBEC pattern G- >A[RD] over G- >A[YN|RC].

Recombinant sequences were identified by a hierarchical test, h-PHI, as previously described [32], which finds all recombinants in a set of sequences (MTCT pair) until the pairwise homoplasy index (PHI) test [33] indicates a probability Pr(PHI) ≥0.05 that homoplasies may have accumulated by other processes than recombination. All such identified recombinant sequences were then evaluated using RIP [57] with a window size of 400 nt, confidence threshold = 0.9, and alignment gaps stripped (very few were present in the data). Potential ancestral sequences were (1) all non-recombinant sequences remaining after h-PHI identification in each MTCT pair, and (2) all sequences except the query sequence in each MTCT pair. The results from using “all” sequences as references were consistent with only using non-recombinant references, but with less power to find significant ancestral assignments because a recombinant ancestor also had its non-recombinant ancestor in the reference set, which would compete in the statistical assignment. Because RIP can only use up to 26 reference sequences, we removed reference sequences based on phylogenetic closeness progressively until n = 26, and also used only the maternal or child references in separate RIP analyses.

Phylogenetic trees were inferred using PhyML [58] under a GTR + I + G substitution model, four category Gamma optimization, with a Bio-NJ starting tree and best of NNI and SPR search, and aLRT SH-like branch support. Phylogenetic networks were inferred using SplitsTree [59] under the default settings.

Molecular clock analyses were performed by ordinary least squares regression of tree-based divergence over time, piece-wise with a potential inflection point at the middle time point for time-dependent evolutionary rate analyses. The rates on each side of the potential inflection point are based on enough time to estimate reliable rates, as HIV-1 populations have been shown to evolve significantly in about 1 month [24]. For analyses of the evolutionary rate as a function of the tree height, the evolutionary rate was estimated in a sliding window moving from the tree root to tips as previously described [23]. The window size was adjusted for each tree so that each window would contain some tips and was moved at 1/10 of the window size in each analysis step.

h-PHI and molecular clock analyses were automated using R [60].

## Additional files

10.1186/s12977-015-0222-0 SplitsTrees and non-recombinant phylogenies of all mother-child transmission pairs. Full taxa names are displayed, and node numbers indicate aLRT support values [61]. The second field in the taxa names indicate age of the respective child, and mother’s age is labeled “x”.
10.1186/s12977-015-0222-0 RIP results of h-PHI identified recombinant child HIV-1 taxa.
10.1186/s12977-015-0222-0 Analysis of potentially advantageous motifs in longitudinally collected env sequences from mother/child pairs.
10.1186/s12977-015-0222-0 Variable region analysis of longitudinally collected env sequences from mother/child pairs.

## Abbreviations

APOBEC: apolipoprotein B mRNA editing enzyme, catalytic polypeptide-like
ARV: antiretroviral
Env: envelope
HIV: human immunodeficiency virus
MTCT: mother to child transmission
PNG: potential n-linked glycosylation site
PHI: pairwise homoplasy index
R5: CCR5 or C–C chemokine receptor 5
RIP: Recombinant Identification Program
RT-PCR: reverse transcription polymerase chain reaction
SGA: single genome amplification
X4: CXCR4 or C–X–C chemokine receptor 4
