# Supplementary figures and images for: Recombination elevates the effective evolutionary rate and facilitates the establishment of HIV-1 infection in infants after mother-to-child transmission
Source: Retrovirology. 2015 Nov 16;12:96. doi: 10.1186/s12977-015-0222-0 (PMC4647327; doi:10.1186/s12977-015-0222-0)

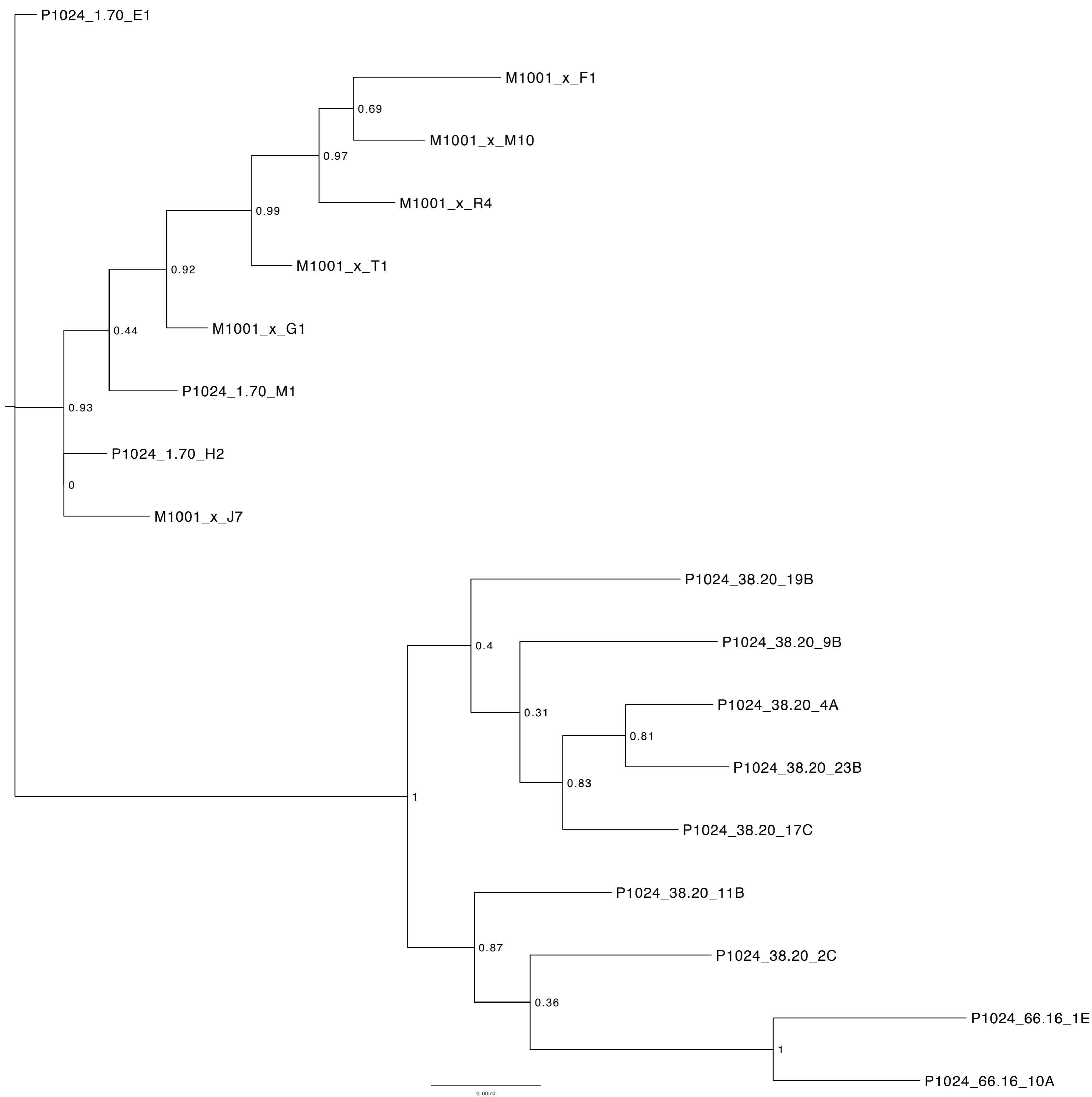

0.0010

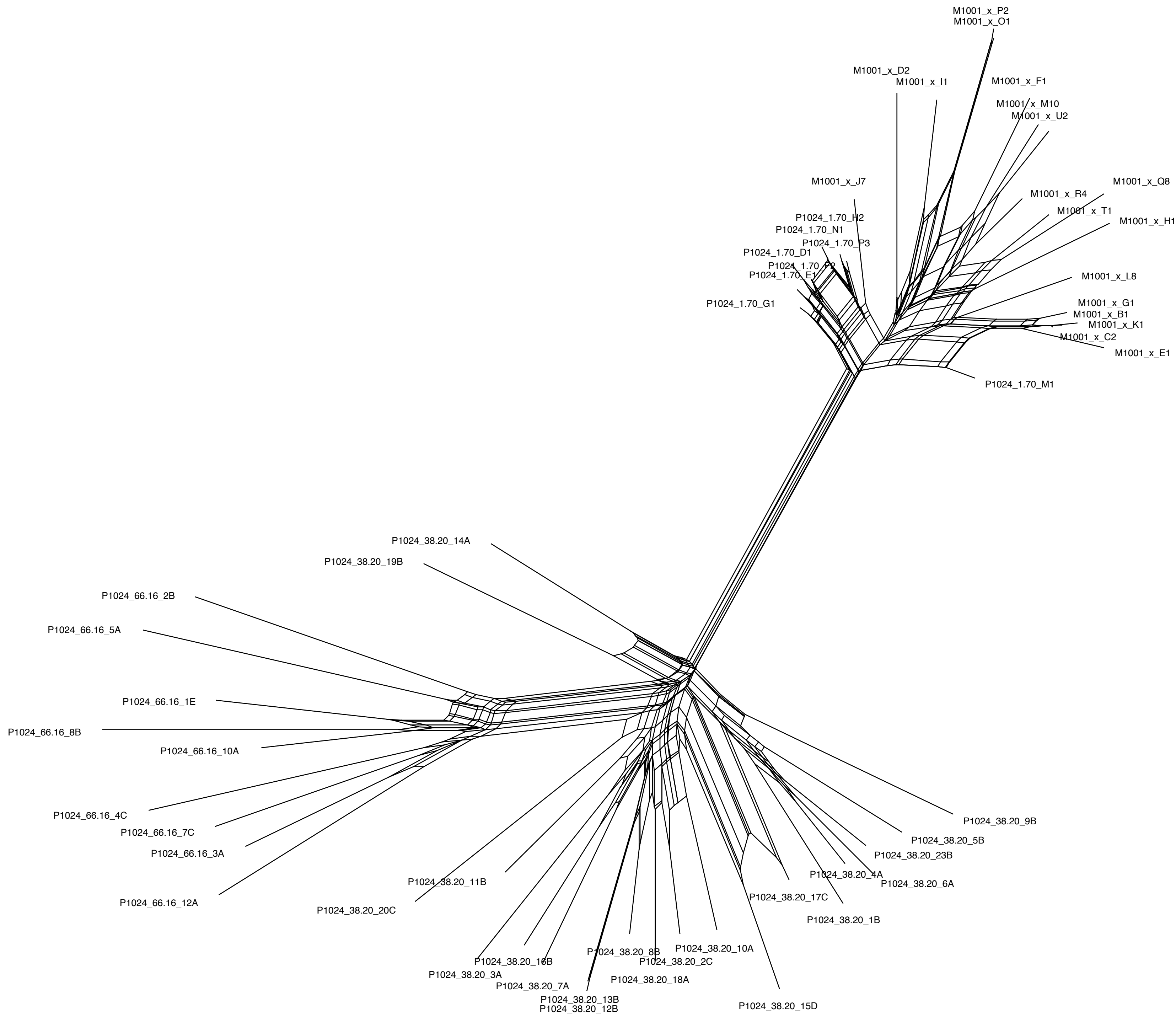

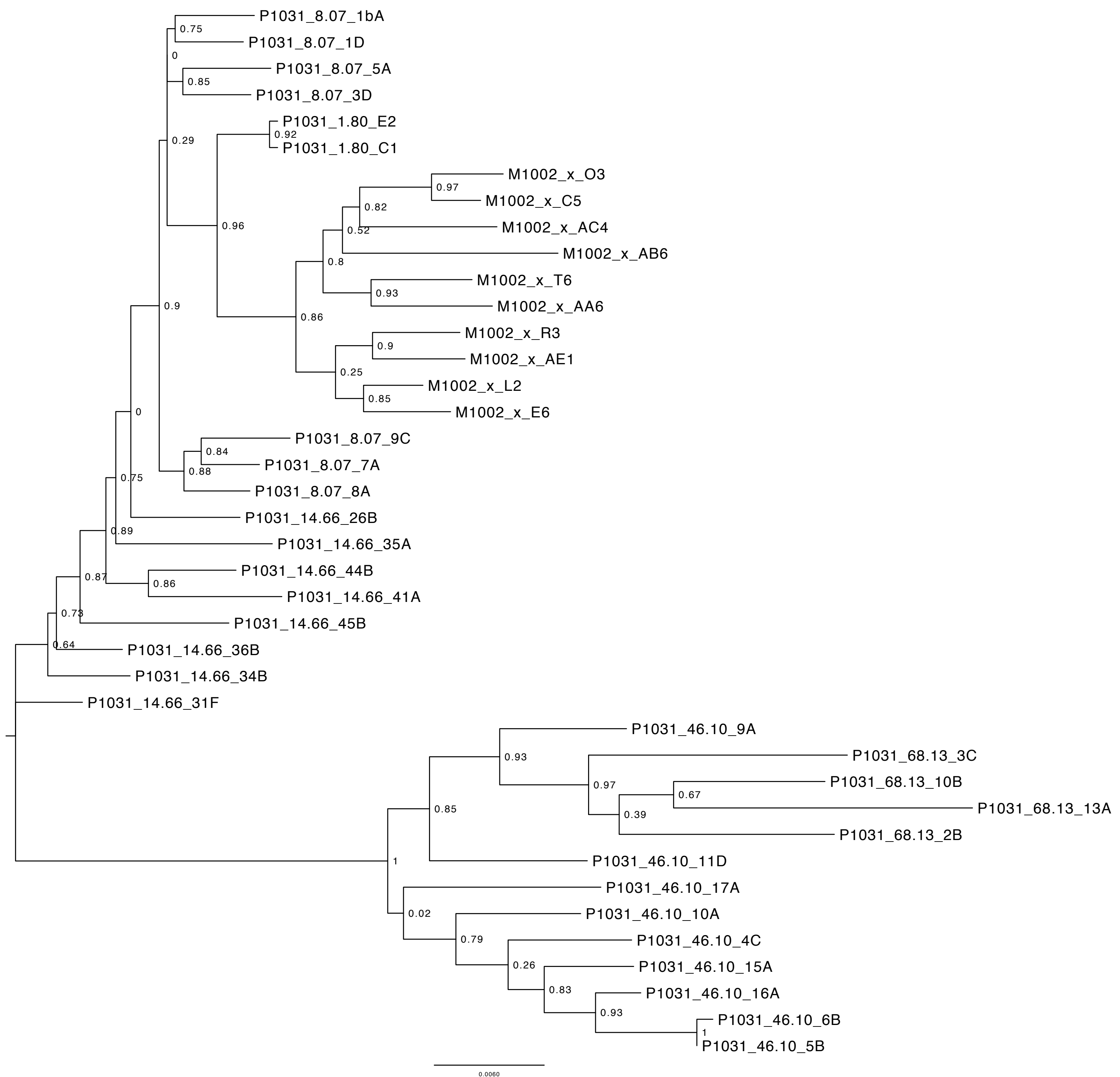

0.0010

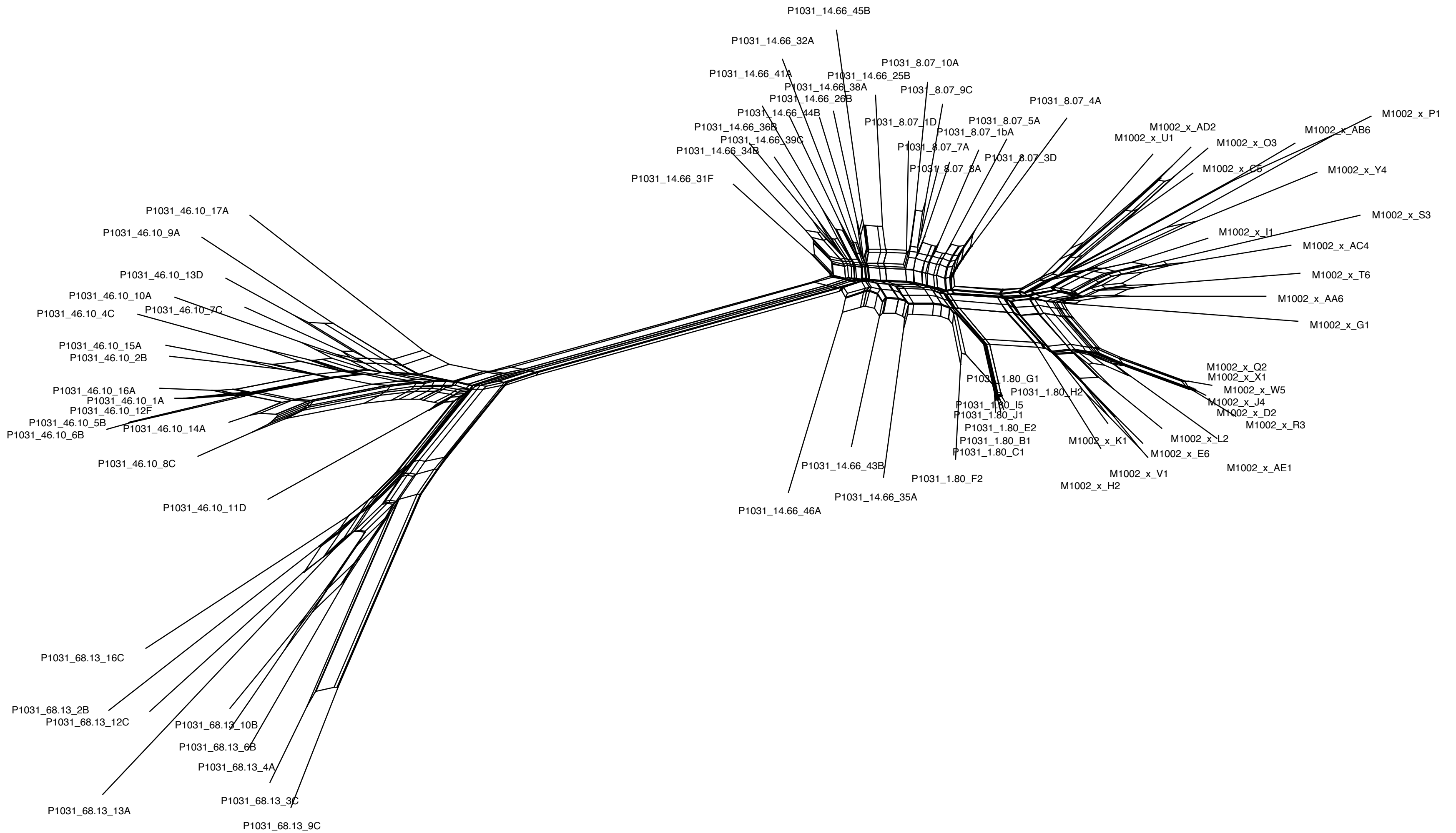

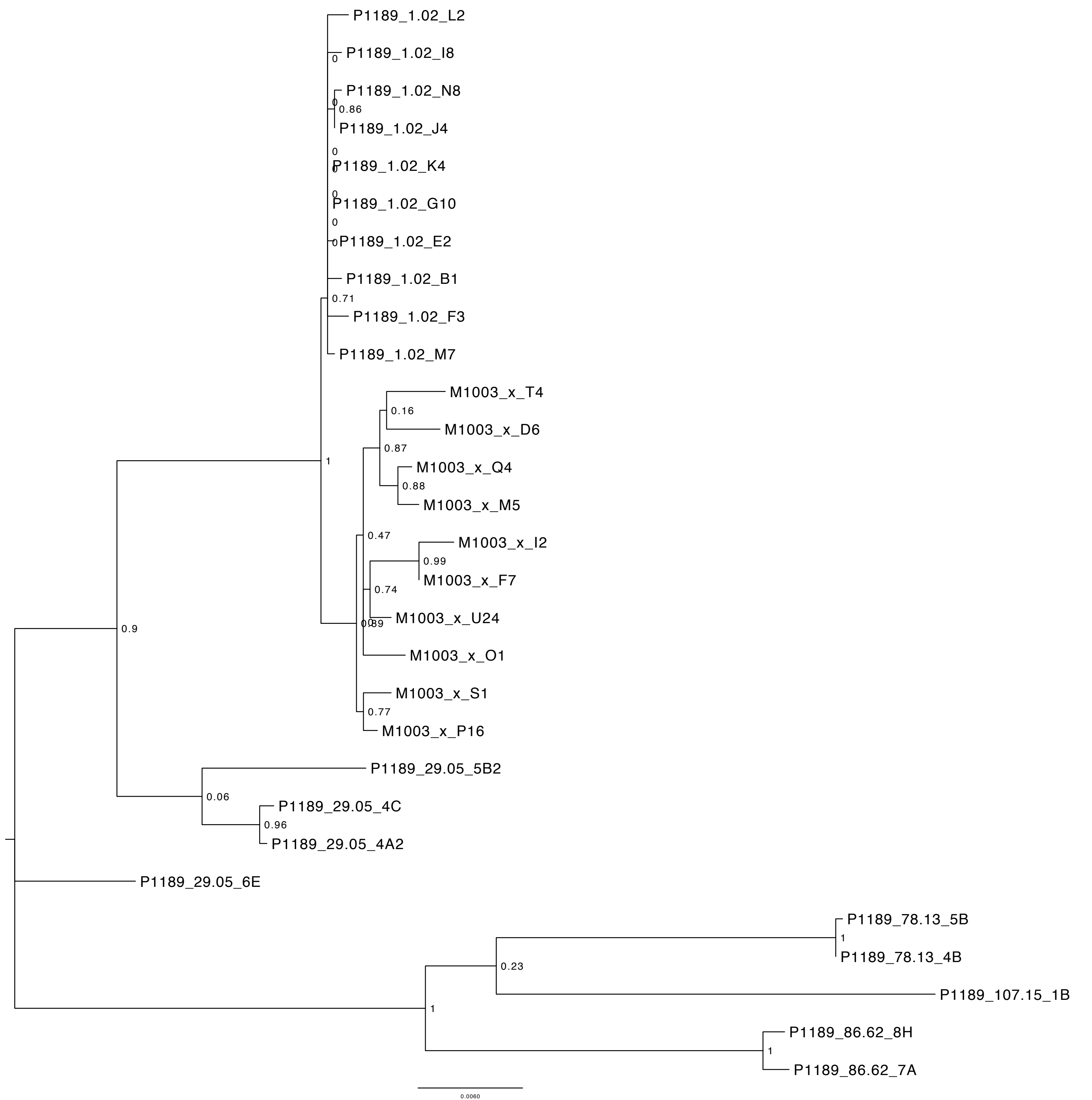

0.0010

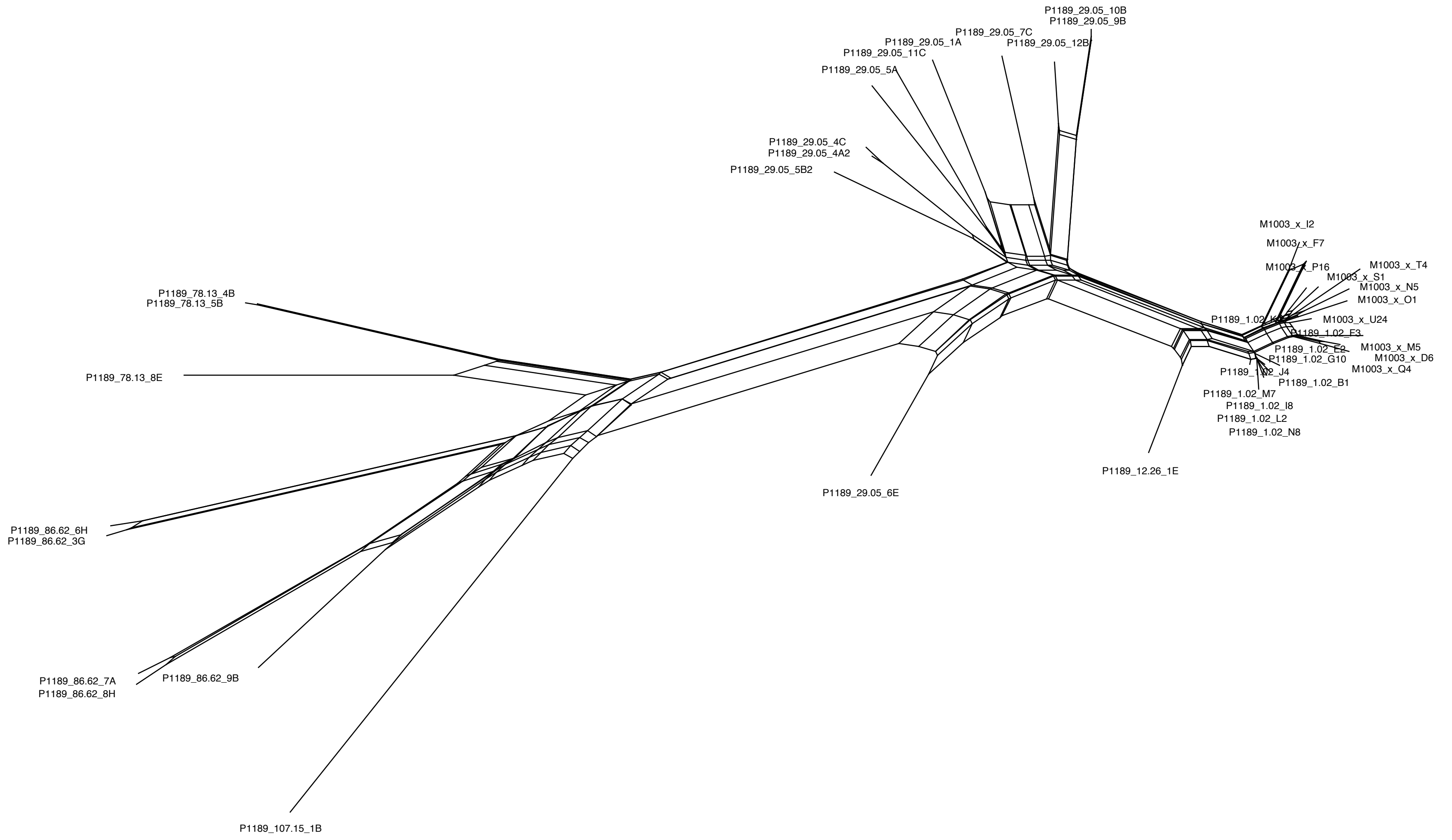

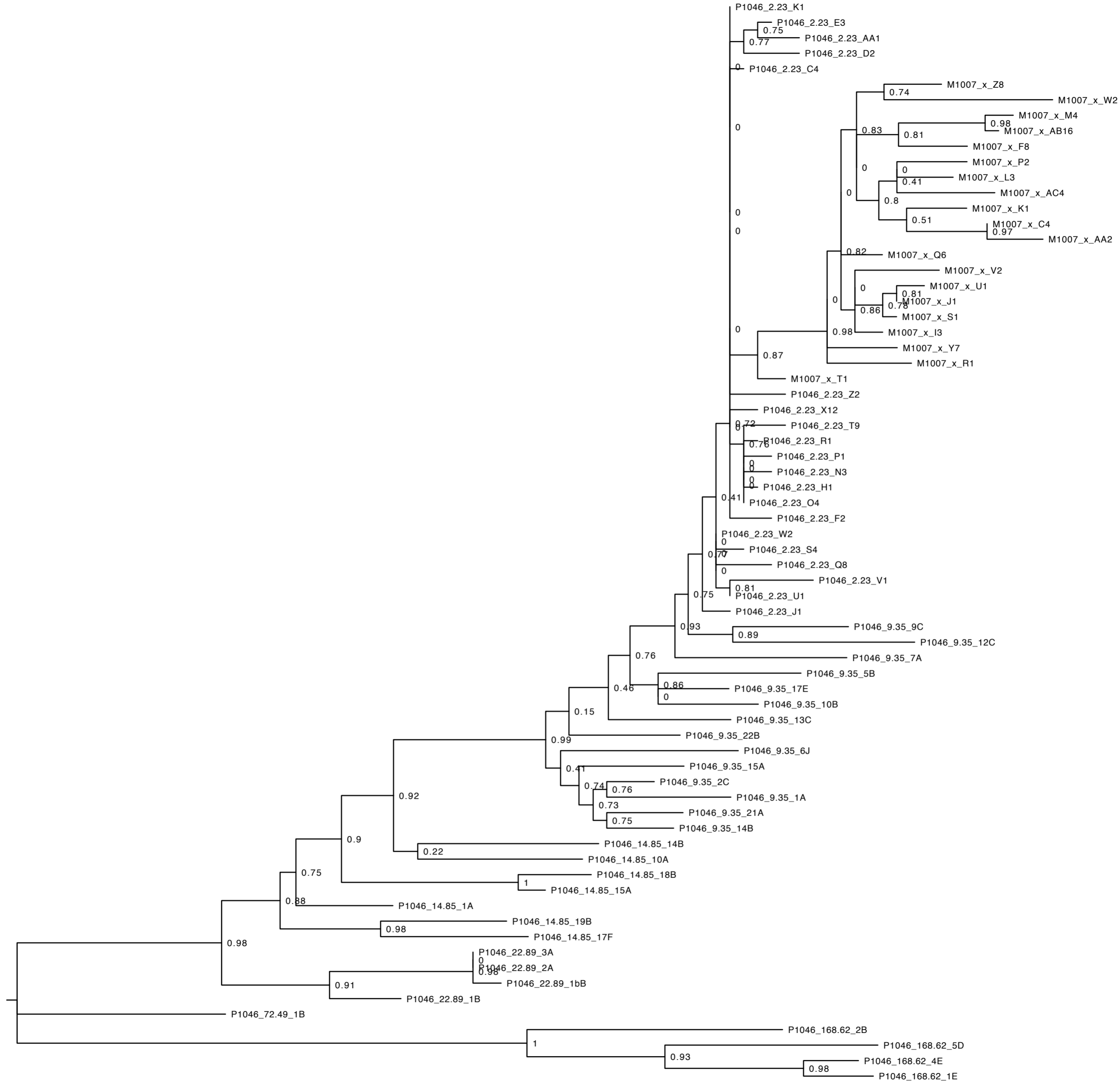

0.0010

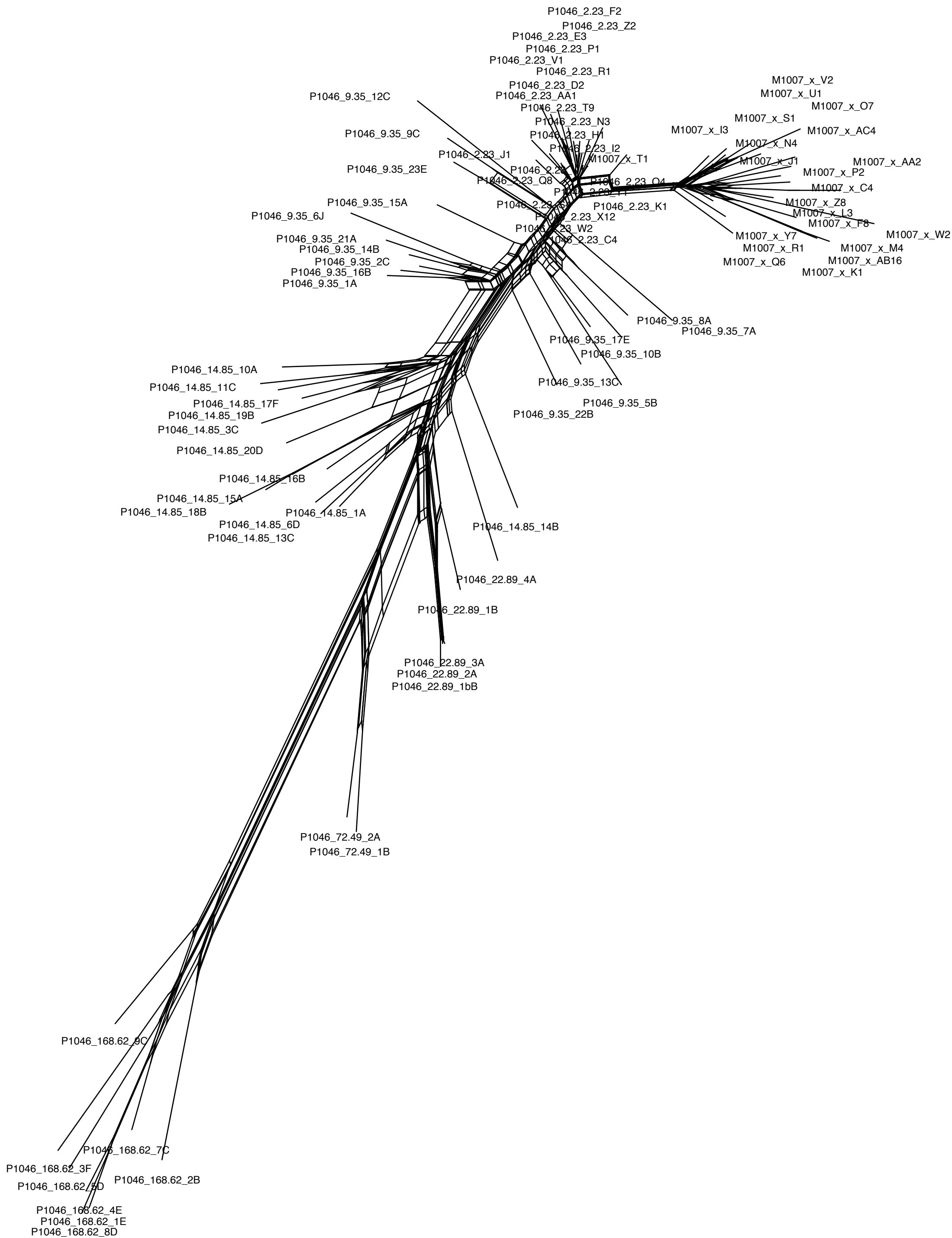

Supplement: Supplementary file 1 — 10.1186/s12977-015-0222-0 SplitsTrees and non-recombinant phylogenies of all mother-child transmission pairs. Full taxa names are displayed, and node numbers indicate aLRT support values [61]. The second field in the taxa names indicate age of the respective child, and mother’s age is labeled “x”. [file 12977_2015_222_MOESM1_ESM.pdf]
