# Supplementary material for: Recombination elevates the effective evolutionary rate and facilitates the establishment of HIV-1 infection in infants after mother-to-child transmission
Source: Retrovirology. 2015 Nov 16;12:96. doi: 10.1186/s12977-015-0222-0 (PMC4647327; doi:10.1186/s12977-015-0222-0)
Supplement: Supplementary file 2 — 10.1186/s12977-015-0222-0 RIP results of h-PHI identified recombinant child HIV-1 taxa. [file 12977_2015_222_MOESM2_ESM.pdf]

Table S1. RIP results of h-PHI identified recombinant child HIV-1 taxa.

Taxon	Related parents
P1024-D1	P1024-E1
P1024-G1	n.s.
P1024-F2	n.s.
P1024-N1	n.s.
P1024-P3	P1024-H2
P1024-10A	P1024-19B, P1024-2C
P1024-18A	P1024-2C
P1024-8B	P1024-2C
P1024-12B	P1024-2C
P1024-13B	P1024-2C
P1024-16B	P1024-2C
P1024-3A	P1024-11B, P1024-2C
P1024-15D	P1024-17C
P1024-6A	P1024-23B. P1024-4A
P1024-5B	P1024-23B
P1024-20C	P1024-17C, P1024-23B
P1024-14A	n.s.
P1024-8B	P1024-10A
P1024-12A	n.s.
P1024-3A	n.s.
P1024-7C	n.s.
P1024-4C	P1024-2C
P1024-5A	n.s.
P1031-10A	n.s.
P1031-4A	n.s.
P1031-12C	n.s.
P1031-16C	P1031-10B, P1031-2B, P1031-11D
P1031-4A	P1031-10B
P1031-6B	P1031-10B, P1031-9A
P1031-9C	P1031-3C
P1031-12F	P1031-16A
P1031-13D	P1031-9A
P1031-14A	n.s.
P1031-1A	P1031-16A
P1031-2B	P1031-15A
P1031-7C	P1031-10A
P1031-8C	P1031-16A
P1031-B1	P1031-E2
P1031-F2	P1031-E2
P1031-G1	P1031-E2
P1031-H2	P1031-E2

P1031-I5	P1031-E2
P1031-J1	P1031-E2
P1031-25B	n.s.
P1031-32A	n.s.
P1031-38A	n.s.
P1031-39C	n.s.
P1031-43B	n.s.
P1031-46A	n.s.
P1189-1E	n.s.
P1189-10B	n.s.
P1189-9B	n.s.
P1189-12B	n.s.
P1189-11C	n.s.
P1189-7C	P1189-5B2
P1189-5A	n.s.
P1189-1A	P1189-5B2
P1189-3G	n.s.
P1189-6H	n.s.
P1189-9B	n.s.
P1189-8E	n.s.
P1046-23E	n.s.
P1046-8A	n.s.
P1046-4A	P1046-1B
P1046-2A	n.s.
P1046-3F	P1046-4E, P1046-5D
P1046-7C	P1046-2B, P1046-4E
P1046-8D	P1046-4E
P1046-9C	P1046-2B, P1046-5D
P1046-I2	n.s.
P1046-Y1	n.s.
P1046-11C	n.s.
P1046-13C	n.s.
P1046-16B	P1046-1A
P1046-20D	P1046-18B
P1046-3C	P1046-17F
P1046-6D	P1046-1A

n.s., no significant parent detected by RIP
