# Supplementary material for: Recombination elevates the effective evolutionary rate and facilitates the establishment of HIV-1 infection in infants after mother-to-child transmission
Source: Retrovirology. 2015 Nov 16;12:96. doi: 10.1186/s12977-015-0222-0 (PMC4647327; doi:10.1186/s12977-015-0222-0)
Supplement: Supplementary file 3 — 10.1186/s12977-015-0222-0 Analysis of potentially advantageous motifs in longitudinally collected env sequences from mother/child pairs. [file 12977_2015_222_MOESM3_ESM.pdf]

**Table S2. Analysis of potentially advantageous motifs in longitudinally collected *env* sequences from mother/child pairs.**

| Patient | Age (months) | 12His | E62D | N92D | Y330H | Q344K | Lack of 156N | Lack of 160N | Lack of 173N | Lack of 197N glycan | Lack of 325N | 328A | Lack of 332N Glycan | Coreceptor Tropism | V2 LDV motif | GPGR | Number of advantageous motifs in recombinant/ non-recombinant sequences |
|---|---|---|---|---|---|---|---|---|---|---|---|---|---|---|---|---|---|
| | advantage: | Increases Env density on virions[1] | Increase possibility of escape from CTL recognition[2] | | | | N glycans are involved in antibody neutralization by PG9/PG16; glycan loss improves resistance to neutralization[3] | | | glycan loss increases CCR5 binding[4] | PGT128 contact[5] | Improves CCR5 binding[6] | PGT128 antibody targets the glycan[7] | CCR5 vs. CXCR4 coreceptor binding[8] | Binding to α4β7 integrin[9] | Broadly neutralizing antibody binding[10] | |
| **M1001** | | 18/0 | 0/18 | 0/18 | 17/1 | 0/18 | 0/18 | 0/18 | 18/0 | 0/18 | 13/5 | 18/0 | 0/18 | R5 | 0/18 | 1/17 | 4.58/4.83 |
| **P1024** | 1.70 | 6/2 | 0/8 | 0/8 | 8/0 | 0/8 | 0/8 | 0/8 | 8/0 | 0/8 | 8/0 | 8/0 | 0/8 | R5 | 0/8 | 0/8 | 4.8/4.67 |
| | 38.20 | 0/21 | 0/21 | 5/16 | 21/0 | 0/21 | 0/21 | 0/21 | 21/0 | 1/20 | 7/14 | 21/0 | 0/21 | R5 | 0/21 | 12/9 | 3.75/3.4 |
| | 66.16 | 0/9 | 0/9 | 3/6 | 9/0 | 0/9 | 0/9 | 0/9 | 9/0 | 0/9 | 3/6 | 9/0 | 0/9 | R5 | 0/9 | 7/2 | 3.5/4 |
| **M1002** | | 24/1 | 5/20 | 0/25 | 25/0 | 0/25 | 0/25 | 11/14 | 25/0 | 0/25 | 25/0 | 25/0 | 0/25 | R5 | 25/0 | 25/0 | 5.71/5.5 |
| **P1031** | 1.80 | 8/0 | 0/8 | 0/8 | 8/0 | 0/8 | 0/8 | 8/0 | 8/0 | 0/8 | 8/0 | 8/0 | 0/8 | R5 | 8/0 | 8/0 | 6/6 |
| | 8.07 | 9/0 | 7/2 | 0/9 | 9/0 | 0/9 | 0/9 | 0/9 | 9/0 | 0/9 | 9/0 | 9/0 | 0/9 | R5 | 9/0 | 9/0 | 5.5/5.86 |
| | 14.66 | 14/0 | 12/2 | 0/14 | 14/0 | 0/14 | 0/14 | 0/14 | 14/0 | 0/14 | 14/0 | 14/0 | 0/14 | R5 | 14/0 | 14/0 | 6/5.75 |
| | 46.10 | 16/0 | 16/0 | 0/16 | 16/0 | 0/16 | 0/16 | 0/16 | 16/0 | 0/16 | 16/0 | 16/0 | 0/16 | R5 | 16/0 | 4/12 | 6/6 |
| | 68.13 | 9/0 | 9/0 | 0/9 | 9/0 | 0/9 | 0/9 | 0/9 | 9/0 | 0/9 | 9/0 | 9/0 | 0/9 | R5 | 9/0 | 3/6 | 6/6 |
| **M1007** | | 22/0 | 22/0 | 0/22 | 22/0 | 0/22 | 0/22 | 0/22 | 22/0 | 0/22 | 22/0 | 22/0 | 0/22 | R5 | 0/22 | 22/0 | 6/6 |
| **P1046** | 2.23 | 22/0 | 22/0 | 0/22 | 22/0 | 0/22 | 0/22 | 0/22 | 22/0 | 0/22 | 22/0 | 22/0 | 0/22 | R5 | 0/22 | 22/0 | 5/5 |
| | 9.35 | 16/0 | 16/0 | 0/16 | 16/0 | 0/16 | 0/16 | 0/16 | 16/0 | 0/16 | 14/2 | 16/0 | 1/15 | R5 | 0/16 | 15/1 | 5.67/6 |
| | 14.85 | 12/0 | 12/0 | 0/12 | 10/2 | 0/12 | 0/12 | 0/12 | 0/12 | 0/12 | 3/9 | 12/0 | 0/12 | R5 | 0/12 | 12/0 | 4/4.17 |
| | 22.89 | 5/0 | 5/0 | 0/5 | 5/0 | 0/5 | 0/5 | 0/5 | 0/5 | 0/5 | 1/4 | 5/0 | 0/5 | R5 | 0/5 | 5/0 | 5/4 |
| | 72.49 | 2/0 | 2/0 | 0/2 | 2/0 | 0/2 | 0/2 | 0/2 | 0/2 | 0/2 | 0/2 | 2/0 | 0/2 | R5 | 0/2 | 2/0 | 4/4 |
| | 168.62 | 8/0 | 8/0 | 0/8 | 8/0 | 0/8 | 0/8 | 0/8 | 0/8 | 0/8 | 0/8 | 8/0 | 0/8 | R5 - one X4 | 0/8 | 5/3 | 4/4 |
| **M1003** | | 11/0 | 0/11 | 0/11 | 11/0 | 0/11 | 0/11 | 0/11 | 11/0 | 0/11 | 11/0 | 11/0 | 0/11 | R5 | 11/0 | 0/11 | 5/5 |
| **P1189** | 1.02 | 10/0 | 0/10 | 0/10 | 10/0 | 0/10 | 0/10 | 0/10 | 10/0 | 0/10 | 10/0 | 10/0 | 0/10 | R5 | 10/0 | 0/10 | -/5 |
| | 12.26 | 1/0 | 0/1 | 0/1 | 1/0 | 0/1 | 0/1 | 0/1 | 1/0 | 0/1 | 1/0 | 1/0 | 0/1 | R5 | 1/0 | 0/1 | 5/- |
| | 29.05 | 10/1 | 0/11 | 0/11 | 11/0 | 0/11 | 0/11 | 11/0 | 11/0 | 0/11 | 11/0 | 11/0 | 0/11 | R5 | 11/0 | 0/11 | 5.86/6 |
| | 78.13 | 3/0 | 0/3 | 0/3 | 3/0 | 0/3 | 0/3 | 0/3 | 3/0 | 0/3 | 3/0 | 3/0 | 0/3 | R5 | 3/0 | 0/3 | 5/5 |
| | 86.62 | 5/0 | 0/5 | 0/5 | 5/0 | 0/5 | 0/5 | 2/3 | 5/0 | 0/5 | 5/0 | 5/0 | 0/5 | R5 | 5/0 | 0/5 | 5.67/5 |

Analysis of potentially advantageous motifs in longitudinally collected *env* sequences is shown, with each residue number referring to HXB2 as a reference sequence. For each patient time point, the number of sequences possessing each motif is shown first, followed by the number of sequences not possessing the motif (yes/no). The final column indicates the average number of potentially advantageous motifs in recombinant sequences, as defined by h-PHI analysis, followed by the average for non-recombinant sequences (recomb/non-recomb). [1] Gnanakaran, 2011; [2] Troyer, 2009; [3] Pancera, 2013; [4] Haim, 2011; [5] Pejchal 2011, Moore 2012; [6] Wang, 1999; [7] Moore, 2012; [8] WebPSSM, http://indra.mullins.microbiol.washington.edu/webpssm/ ; [9] Arthos, 2008; [10] Zolla-Pazner, 2004
