# Supplementary material for: Recombination elevates the effective evolutionary rate and facilitates the establishment of HIV-1 infection in infants after mother-to-child transmission
Source: Retrovirology. 2015 Nov 16;12:96. doi: 10.1186/s12977-015-0222-0 (PMC4647327; doi:10.1186/s12977-015-0222-0)
Supplement: Supplementary file 4 — 10.1186/s12977-015-0222-0 Variable region analysis of longitudinally collected env sequences from mother/child pairs. [file 12977_2015_222_MOESM4_ESM.pdf]

Table S3. Variable region analysis of longitudinally collected *env* sequences from mother/child pairs.

Patient[1]	Age (mos.)	V1			V2			V3			V4			V5		
		Length[2]	Charge[3]	PNGs[4]	Length	Charge	PNGs	Length	Charge	PNGs	Length	Charge	PNGs	Length	Charge	PNGs
**M-1001**	-	32 (31-33)	+2 (0 to 3)	3.5 (3-4)	48 (45-48)	-1 (-3 to -1)	3 (1-3)	35	+4 (4 to 6)	1	34	-4 (-5 to -2)	4 (3-5)	12	-1 (-1 to 0)	1
**P-1024**	1.7	32 (31-32)	+1 (1 to 2)	4 (3-4)	48	-2	3	35	+4	1	34	-5	4	12	0 (-1 to 0)	1
	38.2	27.5 (24-32)	-1 (-1 to 2)	2 (1-3)	43 (39-48)	-1 (-3 to 1)	2 (2-3)	35	+6 (5 to 7)	1	31 (30-32)	-3 (-7 to 0)	3 (3-4)	12 (12-13)	0 (-1 to 0)	2
	66.16	22 (19-27)	0 (0 to 2)	2 (1-2)	41 (39-46)	-1 (-2 to 0)	2	35	5 (4 to 6)	1	30 (30-32)	-1 (-3 to 0)	4 (3-4)	12	1 (0 to 1)	2
**M-1002**	-	31 (26-31)	-2 (-3 to 0)	3 (2-4)	37 (37-41)	+2 (-1 to 3)	2 (1-3)	35	+5	1	30	-1 (-2 to 0)	5 (3-5)	11	0 (-1 to 0)	1 (1-2)
**P-1031**	1.8	31	-2	3	37	+3	1	35	+5	1	30	-1	5 (4-5)	11	-1 (-1 to 0)	1
	8.07	26 (26-31)	-2 (-3 to -2)	2 (2-3)	37	+2	2	35	+5 (4 to 5)	1	30	-1 (-2 to -1)	5	11 (11-13)	-1 (-2 to 0)	1 (1-2)
	11.8	26	-1 (-2 to 0)	2	38 (37-41)	+2 (1 to 2)	3 (2-3)	35	+5	1	30	-1	5 (3-5)	9 (9-13)	0 (-1 to 0)	1 (1-2)
	46.1	26 (26-29)	+1 (1 to 2)	2 (2-3)	41 (39-45)	+1 (1 to 2)	2 (2-4)	35	+5 (4 to 6)	1	30	-1 (-2 to -1)	4 (4-5)	11 (10-11)	0	2 (1-2)
	68.13	25	-1	2 (1-3)	45 (42-45)	+1 (-1 to 1)	4 (3-4)	35	+4 (4 to 5)	1	30	-3 (-4 to 1)	5 (3-5)	10 (10-11)	-1 (-2 to -1)	1 (1-2)
**M-1003**	-	27	-2 (-4 to -1)	2 (1-3)	43	+2	3	35	+5	1	32	-2	5 (4-5)	12	-1 (-1 to 0)	2
**P-1189**	1.02	27	-1 (-1 to 0)	3	43	+2	3	35	+5	1	32	-2	5	12	0	2
	12.26	27	-1	3	50	+2	5	35	+4	1	32	-2	5	12	0	2
	28	22 (22-35)	0 (-2 to 1)	3 (3-5)	50 (43-51)	0 (0 to 2)	4 (2-4)	35	+4	1	30 (28-32)	-2 (-4 to 0)	4 (3-5)	12	-1 (-1 to 0)	3
	78.13	42	0 (0 to 1)	5 (4-5)	43	+2	2	35	+6	1	32 (32-35)	-2 (-4 to -2)	4	12	0	2
	86.62	42 (40-42)	-1 (-3 to -1)	4	43 (41-43)	+2 (1 to 2)	2	35	+6 (5 to 6)	1	35	-4 (-4 to -3)	4	12	0 (-1 to 1)	2 (1-2)
	107.15	41	-1	4	43	+3	2	35	+7	1	30	-1	4	12	0	2
**M-1007**	-	24 (24-31)	-1 (-2 to 0)	2 (2-3)	39	+1 (0 to 1)	2	35	+7 (6 to 7)	1	32	-3	5 (4-5)	13	-1	2
**P-1046**	2.23	31	-2	3	39	+1	2	35	+7	1	32	-3(-4 to -3)	4 (3-4)	13	-1 (-2 to -1)	2 (1-2)
	9.34	24 (19-25)	0 (-2 to 0)	2 (1-2)	39	+1 (1 to 3)	2	35	+6 (6 to 7)	1 (0-1)	32 (28-32)	-3 (-4 to -2)	4 (3-4)	10 (10-13)	-1 (-2 to 0)	1 (1-2)
	14.85	25 (24-25)	0 (-1 to 1)	3 (2-3)	39	+2.5 (1 to 3)	2	35	+6 (5 to 7)	1	32	-3	4 (3-4)	10	-1 (-2 to 0)	1
	22.89	25	-2 (-2 to -1)	3	39	+1	2	35	+6	1	30	-3	4	11 (11-12)	-1	1
	72.49	28	-2.5 (-3 to -2)	3	41	0	2	35	+8	1	30	-2	3.5	14	-2	2
	168.62	36 (36-40)	-2 (-3 to -1)	5 (3-5)	41	+0.5 (0 to 1)	2	35	+7 (6 to 7)	1	30	-2 (-3 to -2)	3.5 (3-4)	13	-2 (-3 to -1)	2

Analysis of variable regions from longitudinally collected *env* sequences is shown, spanning variable regions 1 through 5 (V1-V5) of the *env* sequence.
[1] Patient code. M, maternal; P, child.
[2-4] Length, charge, and number of potential N-linked glycosylation sites (PNGs), respectively, of each variable region, as determined by the Variable Region Characteristics calculator (www.hiv.lanl.gov).
